# Dome-type carcinoma of the colon; a rare variant of adenocarcinoma resembling a submucosal tumor: a case report

**DOI:** 10.1186/1471-230X-12-21

**Published:** 2012-03-08

**Authors:** Masayoshi Yamada, Shigeki Sekine, Takahisa Matsuda, Masayuki Yoshida, Hirokazu Taniguchi, Ryoji Kushima, Taku Sakamoto, Takeshi Nakajima, Yutaka Saito, Takayuki Akasu

**Affiliations:** 1 Pathology Division, National Cancer Center Hospital, Tokyo, Japan; 2 Molecular Pathology Division, National Cancer Center Research Institute, Tokyo, Japan; 3 Endoscopy Division, National Cancer Center Hospital, Tokyo, Japan; 4 Colorectal Surgery Division, National Cancer Center Hospital, Tokyo, Japan; 5 Endoscopy Division, National Cancer Center Hospital, 5-1-1 Tsukiji, Chuo-ku, Tokyo 104-0045, Japan

**Keywords:** Colorectal carcinoma, Gut-associated lymphoid tissue, Dome-type carcinoma

## Abstract

**Background:**

Dome-type carcinoma (DC) is a distinct variant of colorectal adenocarcinoma and less than 10 cases have been described in the literature. Most of the previously reported cases were early lesions and no endoscopic observations have been described so far. We herein report a case of a DC invading the subserosal layer, including endoscopic findings.

**Case presentation:**

A highly elevated lesion in the transverse colon was diagnosed by colonoscopy in a 77-year-old man. The tumor appeared to be similar to a submucosal tumor (SMT), however, a demarcated area of reddish and irregular mucosa was observed at the top of the tumor. There were no erosions or ulcers. Laparoscopic-assisted right hemicolectomy was performed and pathological examination revealed a well-circumscribed tumor invading the subserosal layer. The tumor was a well-differentiated adenocarcinoma associated with a dense lymphocytic infiltration and showed expansive growth. The overlying mucosal layer showed high-grade dysplasia.

**Conclusion:**

The present lesion was diagnosed as a DC of the colon invading the subserosal layer. Because the association of mucosal dysplasia is common in DCs, the detection of dysplastic epithelium would be important to discriminate DCs from SMTs.

## Background

Dome-type carcinoma (DC) is a rare variant of colorectal adenocarcinoma that is characterized by well or moderately differentiated histology, expansive growth, and dense lymphoid stroma [1]. Since Jass *et al. *[1,2] reported this lesion as a distinct variant of adenocarcinoma, less than 10 cases have been reported and most of them are early lesions limited to the submucosal layer [3,4]. Based on the phenotypical features of DCs, including the intimate association with lymphoid tissue, the presence of intraepithelial B-lymphocytes and the lack of goblet cells, DC has been suggested to derive from M-cells of the gut-associated lymphoid tissue [1].

We herein report a case, along with the endoscopic findings, of a DC invading the subserosal layer.

## Case presentation

**Figure 1:**
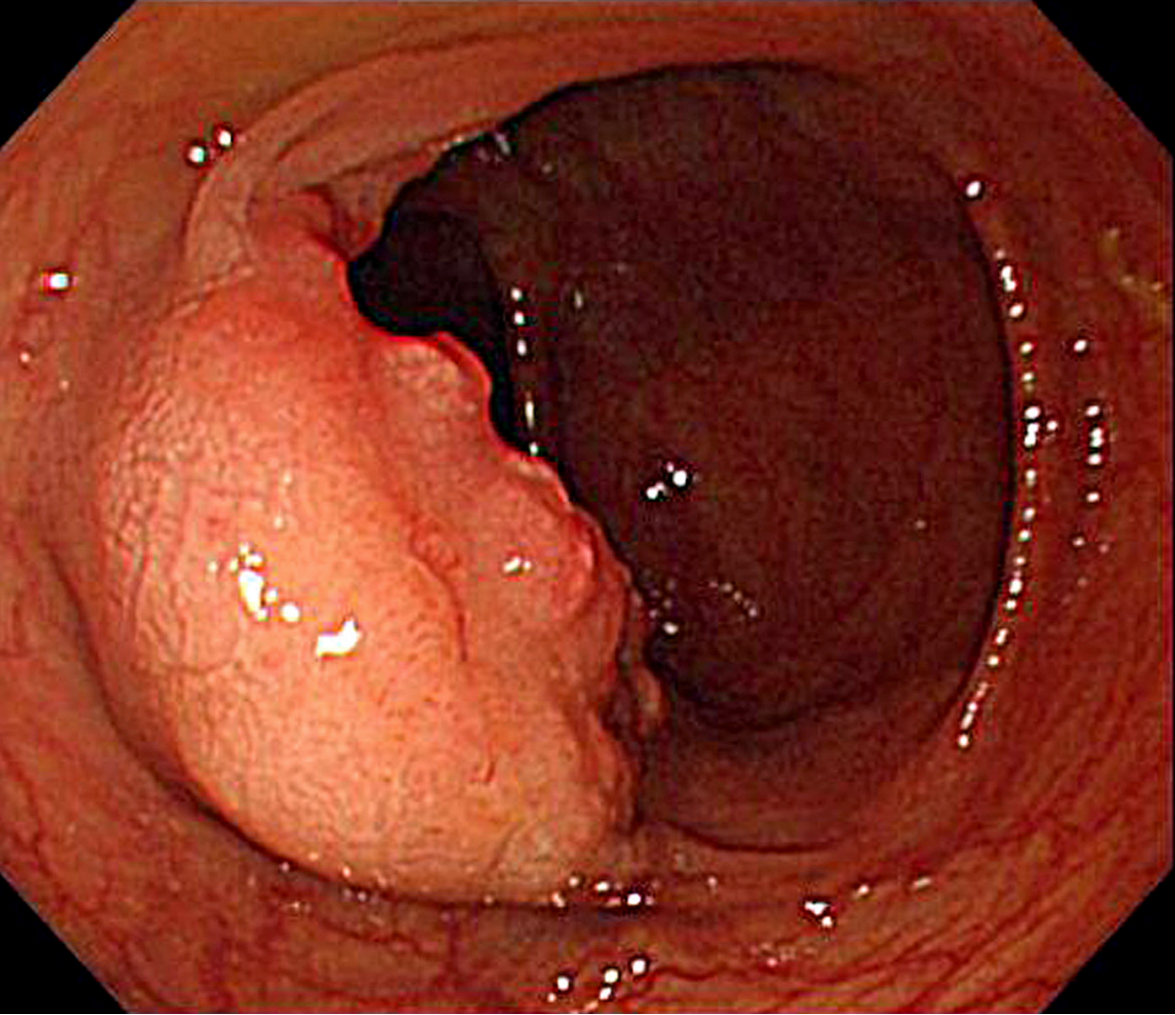
**Conventional endoscopic image showing a submucosal tumor-like lesion of 30 mm in diameter in a 77-year-old man**. A reddish rough mucosa can be seen on the top.

**Figure 2:**
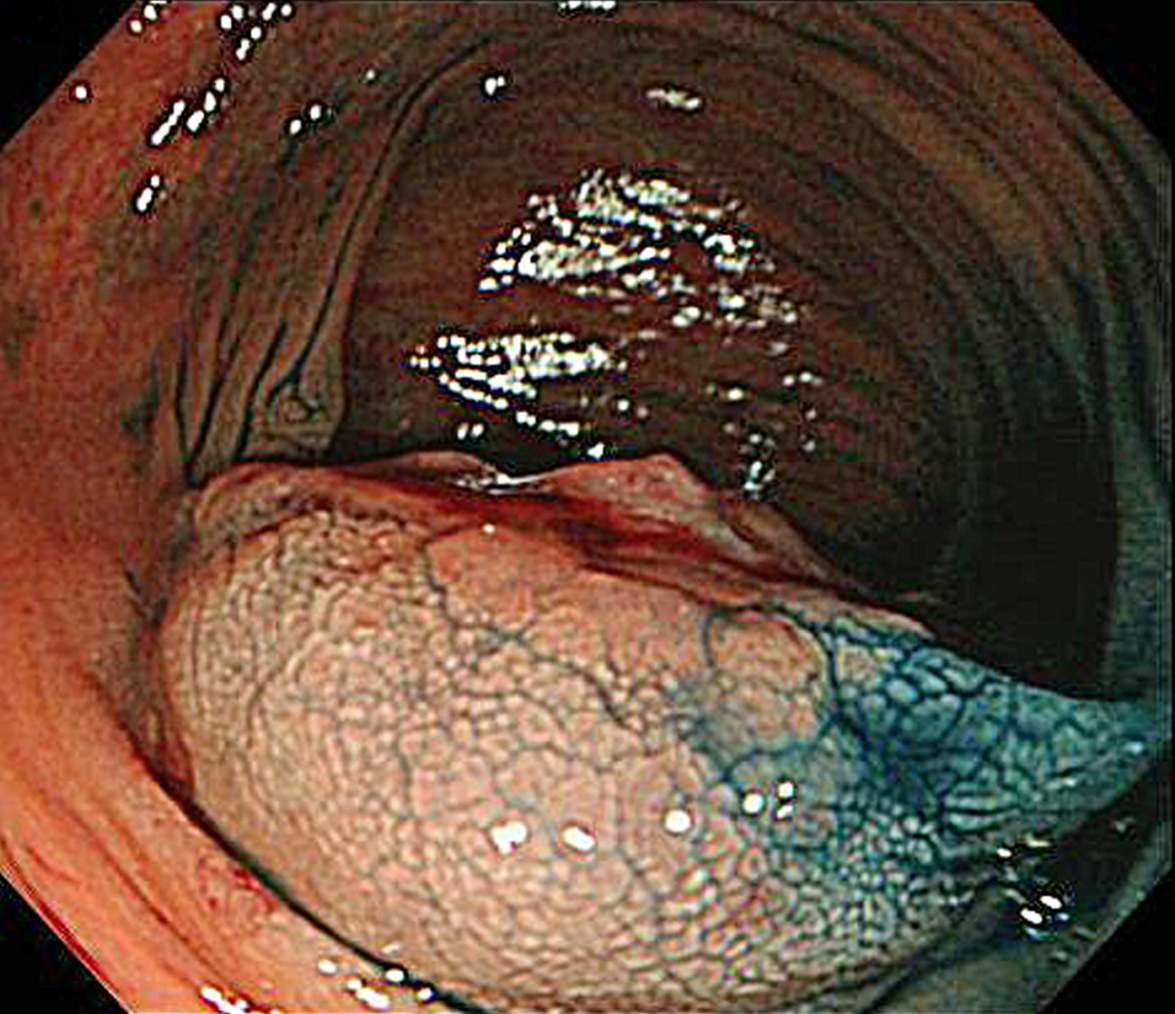
**Endoscopic image after spraying with indigo carmine dye**. The base of the tumor is covered with non-neoplastic mucosa.

**Figure 3:**
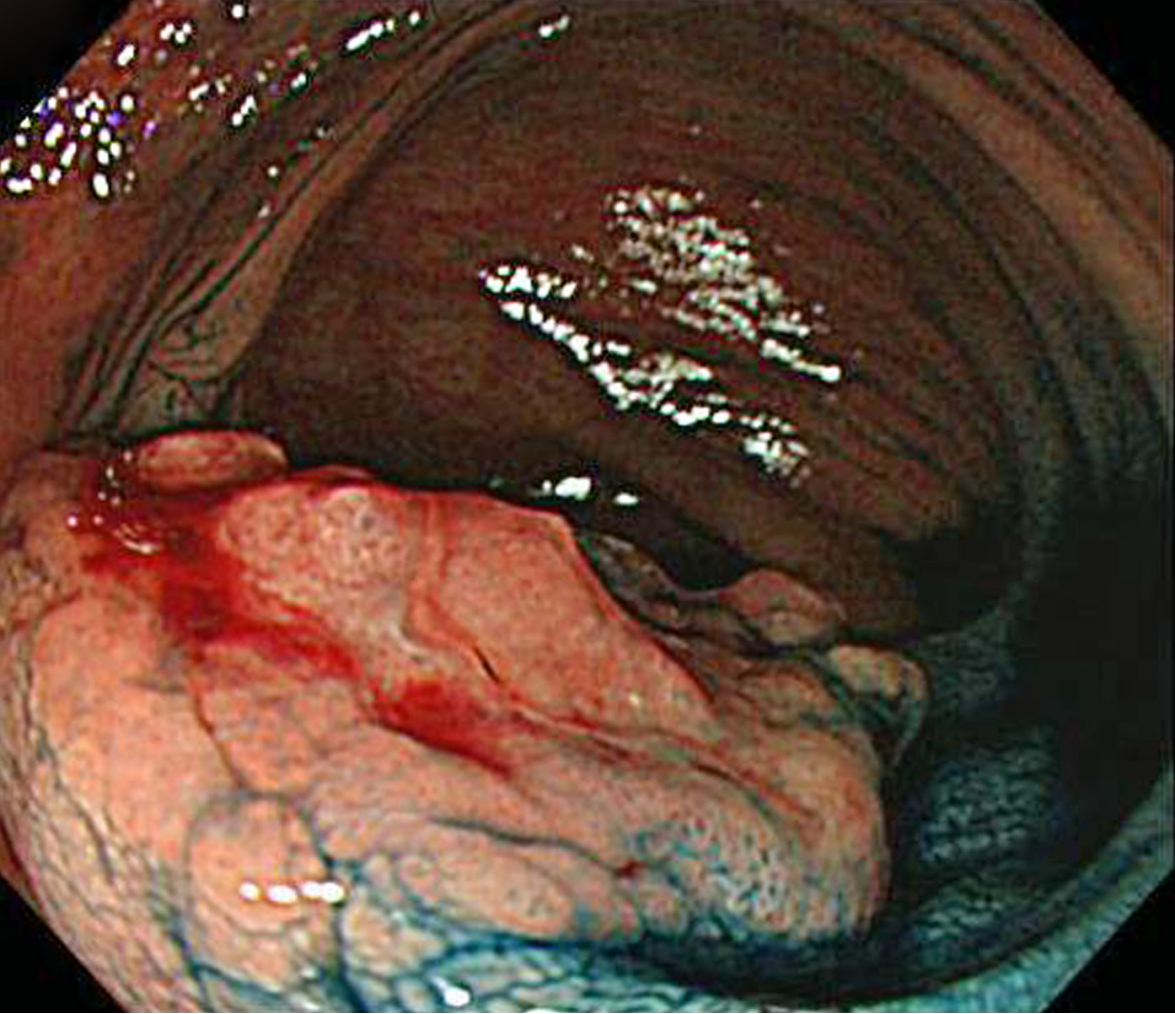
**The top of the tumor, showing a well demarcated irregular mucosa**.

A 77-year-old man suffered abdominal discomfort and underwent a total colonoscopy. The colonoscopy identified a highly elevated lesion, 30 mm in diameter, in the transverse colon (Figure 1). The tumor appeared to be similar to a submucosal tumor (SMT) with a sharply raised edge and a bridging fold. Examination with indigo carmine dye showed that the base of the lesion was covered with normal mucosa (Figure 2). However, a demarcated area of reddish and irregular mucosa was observed at the top of the tumor (Figure 3). There were no erosions or ulcers. The biopsy specimen taken from the top of the lesion revealed well-differentiated adenocarcinoma. Finally, the lesion was diagnosed as adenocarcinoma confined to the transverse colon and a laparoscopic-assisted right hemicolectomy was performed.

**Figure 4:**
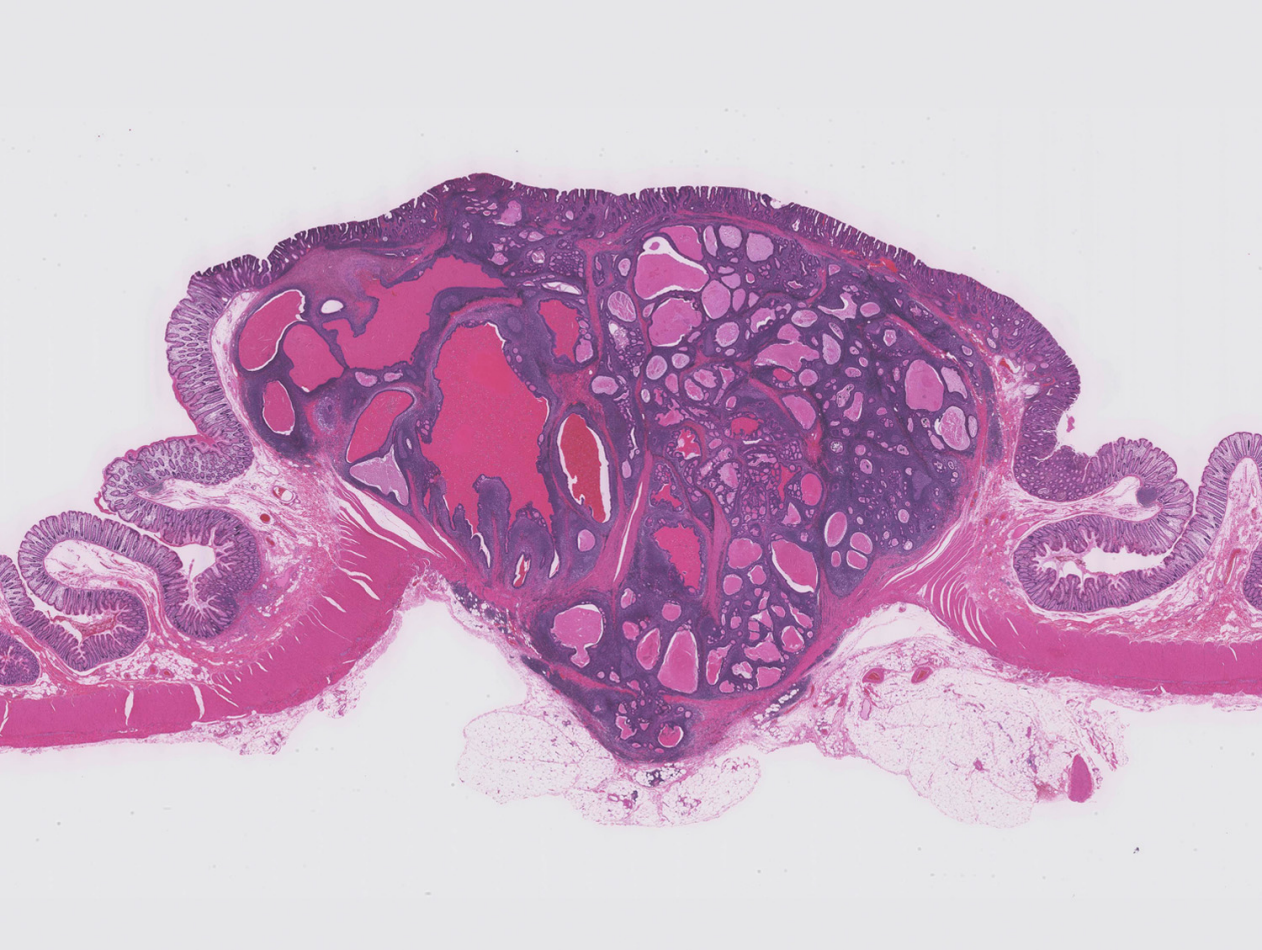
**Panoramic view of the tumor described. A well-demarcated tumor grows into the subserosal layer (H&E)**.

**Figure 5:**
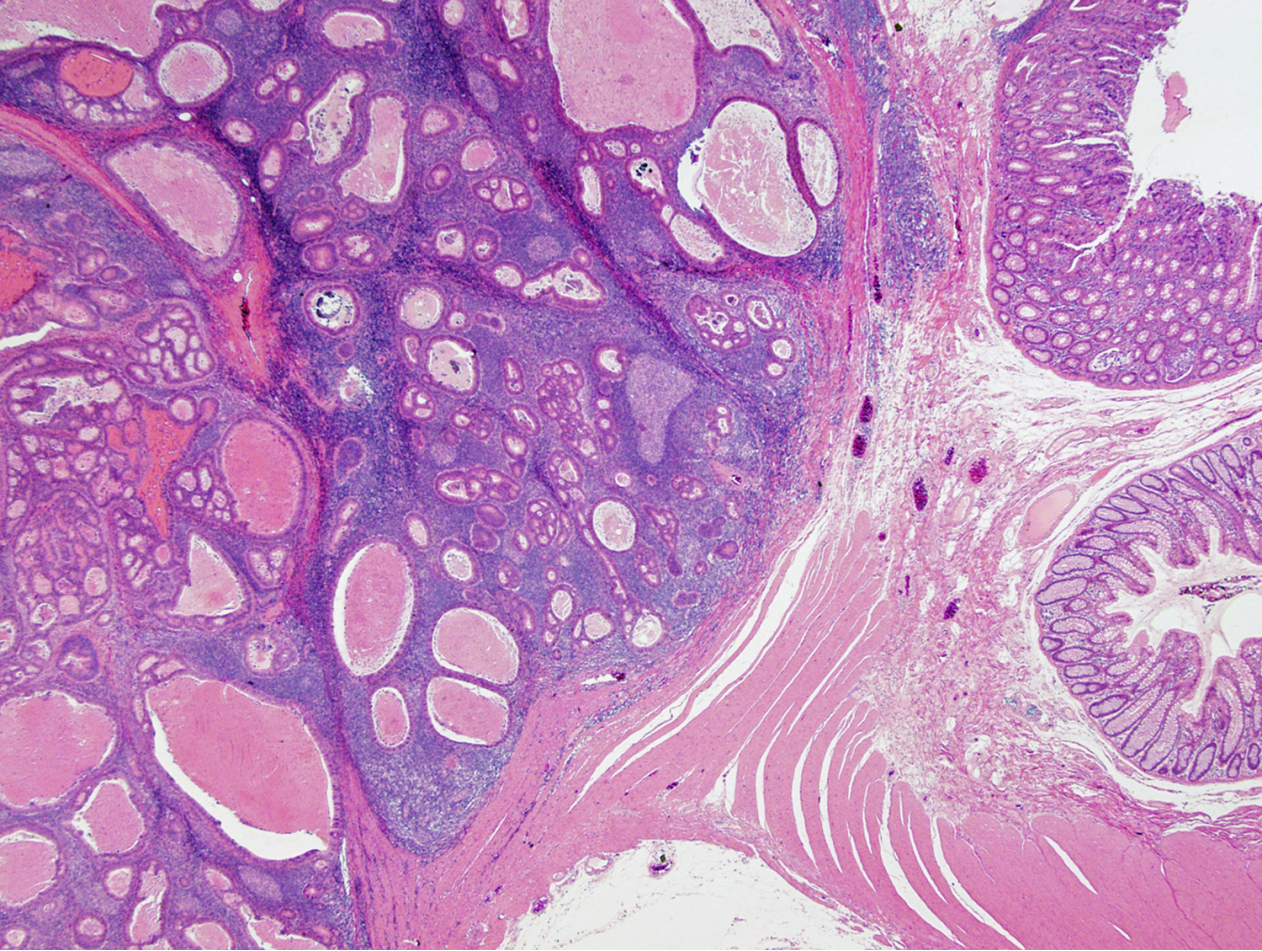
**The tumor associated with lymphoid stroma showing expansive growth. No desmoplastic stroma is observed (H&E, orig. mag. ×12.5)**.

**Figure 6:**
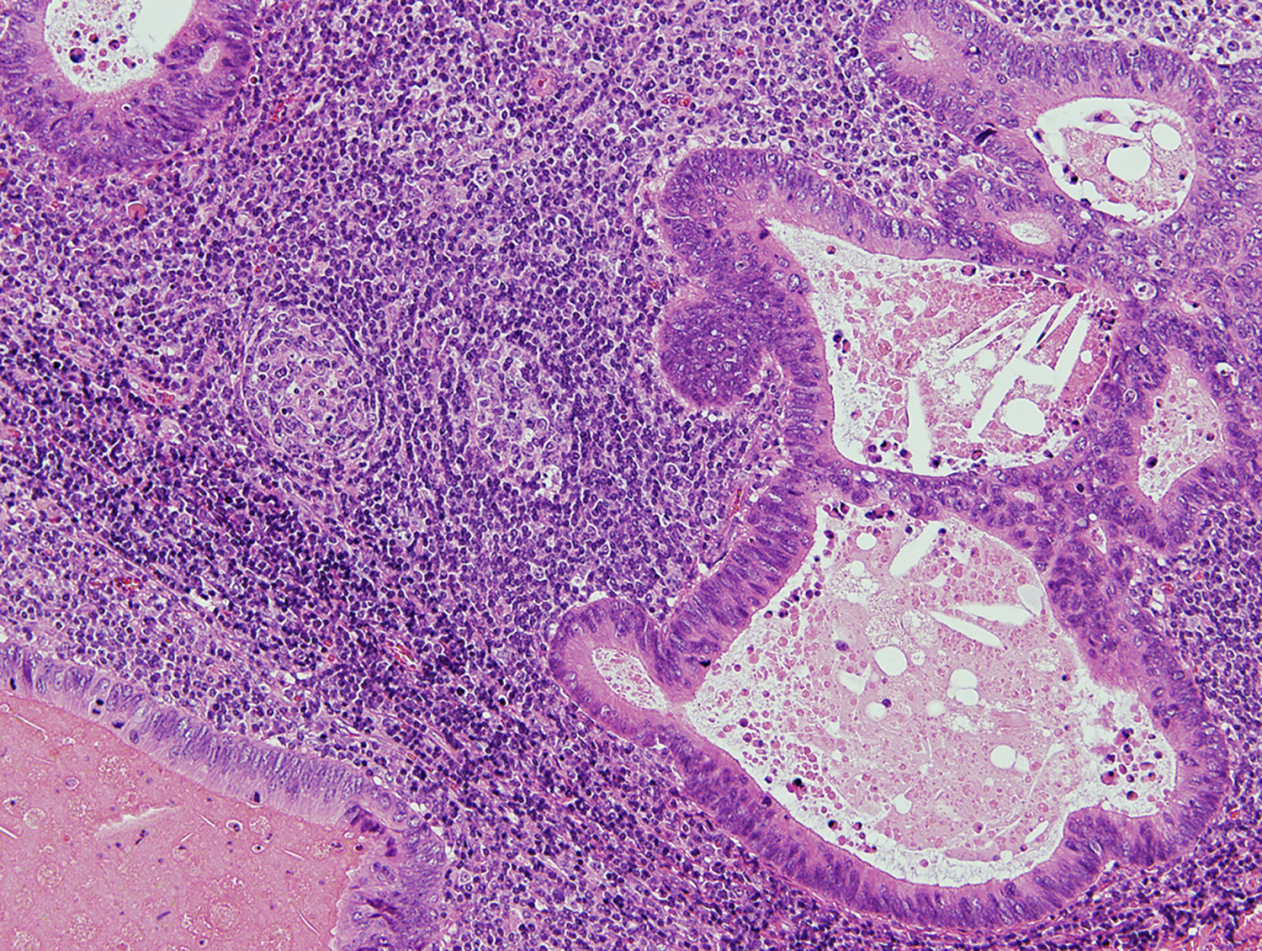
**The tumor is a well differentiated adenocarcinoma surrounded by dense lymphoid tissue with follicles**. Neoplastic glands contain eosinophilic necrotic debris (H&E, orig. mag. ×100).

**Figure 7:**
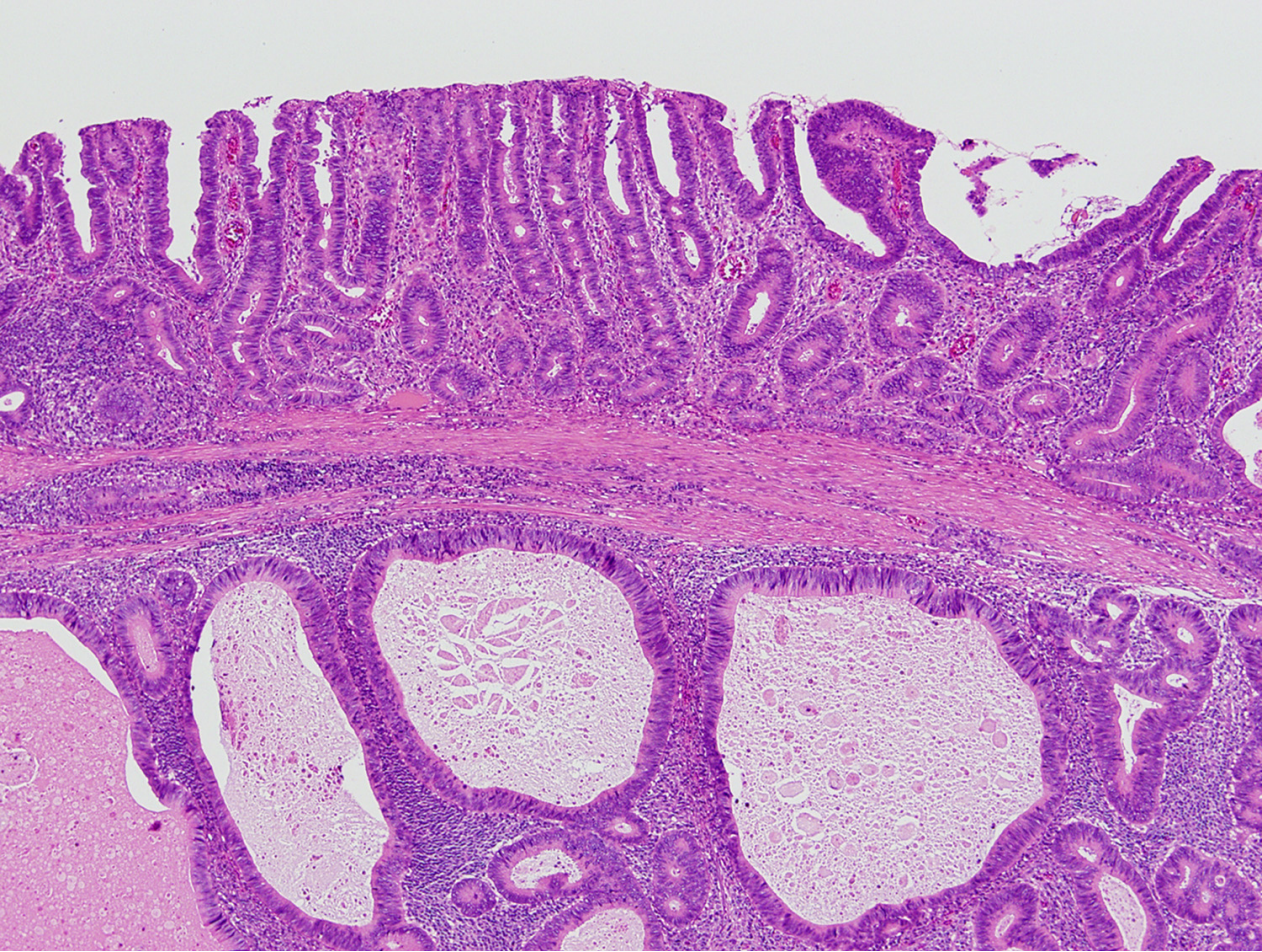
**Overlying mucosa shows high-grade dysplasia**. Invasive adenocarcinoma associated with prominent lymphoid stroma was observed in the submucosal layer. Note the intact muscularis mucosae (H&E, orig. mag. ×40).

Pathological examination revealed a well-circumscribed tumor invading the subserosal layer (Figure 4). The tumor was a well-differentiated adenocarcinoma associated with a dense lymphocytic infiltration. The tumor showed expansive growth and no desmoplastic stroma was seen (Figure 5). Many of the tumor glands were cystically dilated and contained eosinophilic debris (Figure 6). The lymphoid stroma surrounding the neoplastic glands contained numerous germinal centers. The overlying mucosal layer showed high-grade dysplasia (Figure 7). Immunohistochemically, tumor cells were positive for 4 mismatch repair proteins (MLH1, PMS2, MSH2, MSH6), suggesting microsatellite stable phenotypes. In situ hybridization for Epstein-Barr virus (EBV) -encoded small RNA-1 was negative. No metastasis was detected in any of the 19 dissected lymph nodes. One and a half years after the resection, no recurrence was detected by follow up computed tomography or endoscopic examination.

## Conclusions

Jass *et al. *[1,2] reported 3 cases of "Adenocarcinoma of colon differentiating as dome epithelium of gut-associated lymphoid tissue" as a distinct variant of colon cancer. The reported lesions were characterized by well and/or moderately differentiated histology, expansive growth, confinement to an aggregate of lymphoid tissue, and cystically dilated tumor glands containing an abundance of necrotic debris. Because of the intimate relationship between the malignant epithelium and lymphoid tissue, they suggested that the tumor might be arising from the dome epithelium overlying gut-associated lymphoid tissue. After similar tumors were reported, the term DC was established [3-8].

Generally, prominent lymphocytic infiltration is known as a feature of colorectal cancers with a microsatellite instability-high phenotype and tumors with EBV infection. However, the present case, and the majority of the previously reported DCs, did not show evidence for microsatellite instability, as examined by either microsatellite instability test or immunohistochemistry for mismatch repair proteins, and EBV infection [9]. The lack of evidence for microsatellite instability and EBV infection is consistent with the concept that lymphoid infiltration associated with DCs reflects the nature of their tissue of origin, which is the dome epithelium.

All but one previously reported DCs were early cancers limited to the submucosal layer [3]. It has been suggested that advanced DC is rare because DC might eventually progress to usual-type adenocarcinoma [7]. Consistent with this idea, 4 of 9 previously reported DCs, including one lesion that invaded the muscularis propria, were associated with a usual-type adenocarcinoma component that is characterized by the association with a desmoplastic reaction and the lack of lymphoid stroma [2,4,6,7]. However, the present case indicates that, in rare instances, DC can deeply invade the bowel wall in the absence of progression to usual-type adenocarcinoma.

Endoscopically, the present case resembled SMT, reflecting the expansive growth of the tumor. However, while the base of the lesion was covered with non-neoplastic mucosa, an area of mucosal dysplasia could be endoscopically detected on the top of the lesion, and a biopsy taken from this area allowed a diagnosis of adenocarcinoma. Because the previously reported DCs also lacked erosion or ulceration and were associated with mucosal dysplasia [2-4,7], the detection of dysplastic epithelium would be important to discriminate DCs from SMTs.

Even though the current classifications do not recognize DC as a distinct histological subtype, the present and previous reports illustrated peculiar histological and clinical characteristics of DC. Further accumulation of cases and phenotypical characterization, including the potential relationship to M-cells, may establish DC as a distinct subtype of colorectal adenocarcinoma.

## Consent

Written informed consent was obtained from the patient for publication of this case report and any accompanying images. A copy of the written consent is available for review by the editor-in-chief of this journal.

## Abbreviations

DC: Dome-type carcinoma; SMT: Submucosal tumor; EBV: Epstein-Barr virus.

## Competing interests

The authors declare that they have no competing interests.

## Authors' contributions

MY for design and drafting of the manuscript; Dr. SS for the concept and the revision of the manuscript and the pathological diagnosis; Dr. TM for the revision of the manuscript and the supervision; Drs. MY, HT, and RK for the pathological diagnosis; Drs. TS, TN and YS for the endoscopic diagnosis; Dr. TA for the surgical treatment. All authors read and approved the final manuscript.

## Pre-publication history

The pre-publication history for this paper can be accessed here:

http://www.biomedcentral.com/1471-230X/12/21/prepub
